# Supplementary material for: Multi-Responsive Sensor Based on Porous Hydrogen-Bonded Organic Frameworks for Selective Sensing of Ions and Dopamine Molecules
Source: Molecules. 2022 Dec 9;27(24):8750. doi: 10.3390/molecules27248750 (PMC9781585; doi:10.3390/molecules27248750)
Supplement: Supplementary file 1 [file molecules-27-08750-s001.zip › molecules-2073740-supplementary.pdf]

Multi-responsive sensor based on porous hydrogen-bonded organic frameworks for selective sensing of ions and dopamine molecules

1. Experimental Details

1.1. Materials and general methods

All chemicals were obtained commercially and used without further processing and purification. The Powder X-ray diffraction (PXRD) patterns of HOF-TCBP were measured by X-ray diffractometer（D2 PHASER） with Cu Kα radiation (λ=1.54056 Å) at a scan rate of 5°/min and a scan range of 5° – 50°. Field emission scanning electron microscopy (FE-SEM) images were obtained by MODEL SU8010, Hitachi. The PL spectrum were obtained by F4600 fluorescence spectrometer. FT-IR was carried out on a Nicolet iS50 spectrometer from Thermo Fisher Scientific. UV-Vis absorption spectrum was recorded on Shimadzu UV-3600 spectrometer.

1.2. Synthesis of HOF-TCBP

Add 3,3′,5,5′-tetrakis-(4-carboxyphenyl)-1,1′-biphenyl (H_4TCBP) (100 mg, 0.158 mmol) and DMF (0.7 mL) to a 20 mL vial. After 10 minutes of sonication, let stand for several days, and let the liquid slowly evaporate and crystallize. Then, add 0.5 mL of DMF to dissolve and 5 mL of acetone for anti-dissolving, mix well, and filter. Finally, it was washed several times with acetone and air-dried to obtain a white powder.

1.3. Metal ion sensing

In metal ion sensing experiments, a 10 mg HOF-TCBP powder sample was weighed, then dispersed into 200 mL of ethanol solvent, and after sonication for 30 min, a HOF-TCBP suspension was obtained. Prepare a series of nitrate solutions $A(NO_3)_x$ (10^{-3}M, A = Ca^{2+}, K^+, Cd^{2+}, Al^{3+}, Co^{2+}, Zr^{4+}, Sc^{3+}, Cu^{2+}, Fe^{3+}, Cr^{3+}), according to the titration method, gradually add to 2 mL of the HOF-TCBP suspension, and then measure the fluorescence spectrum.

1.4. Anion sensing

For the anion sensing experiments, the sodium salt aqueous solutions of Ac^-, Br^-, Cl^-, CO_3^{2-}, F^-, HPO_4^{2-}, SiO_3^{2-}, SO_3^{2-}, SO_4^{2-}, $Cr_2O_7^{2-}$ with a concentration of 10^{-3} M were first prepared,and then different amounts of the above stock solutions were added to 2 mL HOF-TCBP suspension, respectively, and the luminescence data were collected after standing overnight. In the anti-interference experiment of $Cr_2O_7^{2-}$, 230 μL of another nine anion solutions were added to 2 mL HOF-TCBP suspension, and then the $Cr_2O_7^{2-}$ solution was added incrementally, and the luminescence data were collected after standing overnight.

1.5. Dopamine sensing

Prepare a DA stock solution at a concentration of 10 mM. Meanwhile, the HOF-TCBP samples were dispersed in phosphate buffer solution (5 mg/100 mL) with pH 2 - 6, respectively, and ultrasonically treated for 30 min. Then 2 mL HOF-TCBP suspensions with different pH were taken respectively, and DA solution was added dropwise in order to measure the fluorescence spectrum. In addition, seven solutions were also prepared at a concentration of 10 mM, cysteine (Cys), phenylalanine (Phe), glutamic acid (Glu), urea (Urea), glucose (Glc), tryptophan (Trp) and aspartic acid (Asp) were added to 2 mL HOF-TCBP suspension at pH = 6, respectively, and the fluorescence was measured for selectivity experiments. The fetal bovine serum was diluted 30-fold before the measurement of the real sample fetal bovine serum.

2. Figures

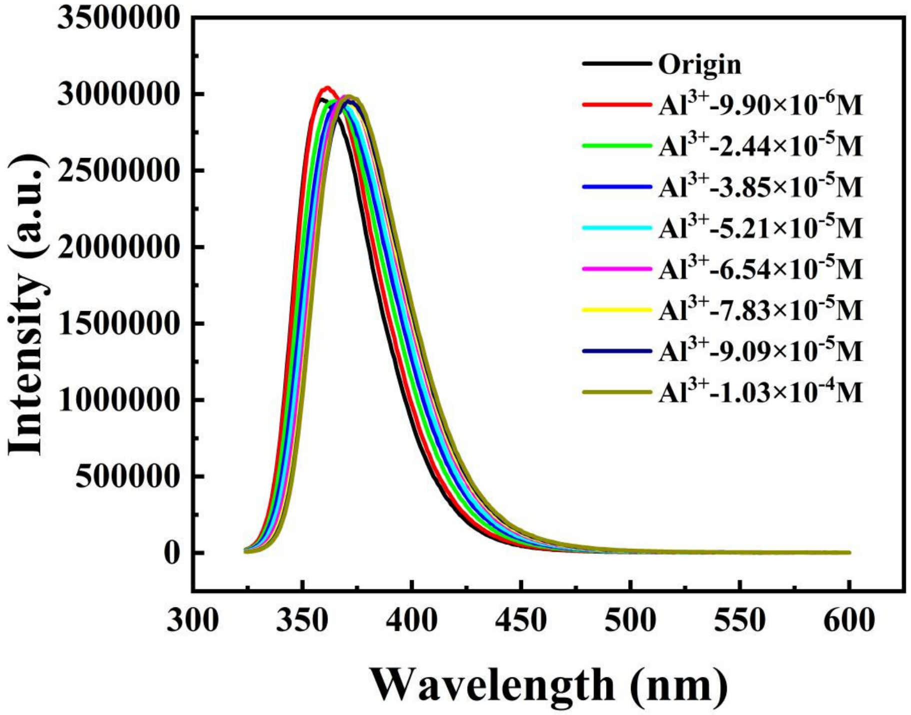

Figure S1. The PL spectrum of HOF-TCBP with different amounts of Al^{3+}.

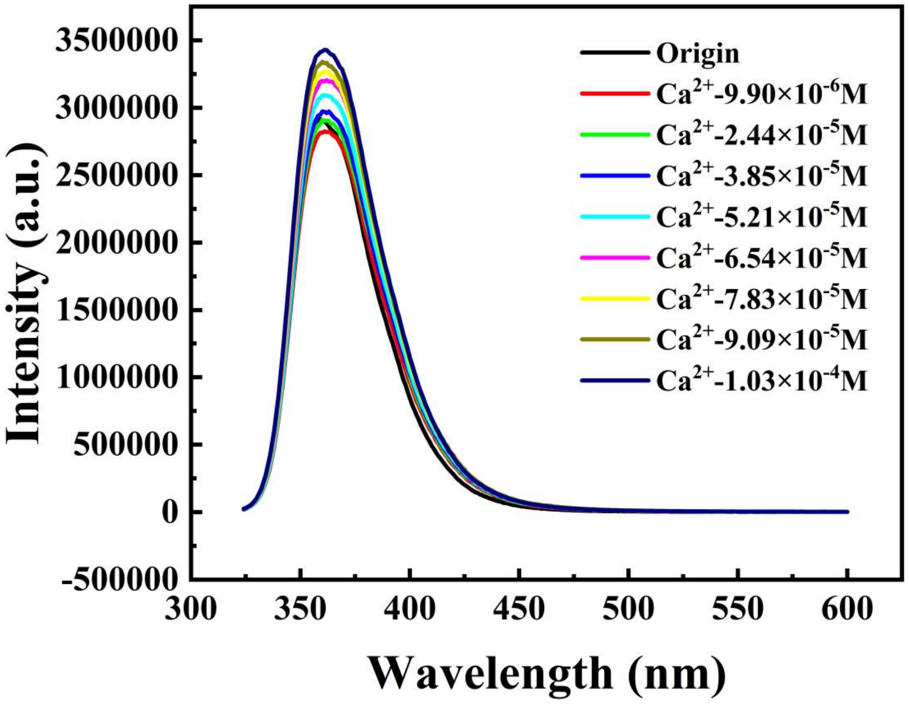

Figure S2. The PL spectrum of HOF-TCBP with different amounts of Ca^{2+}.

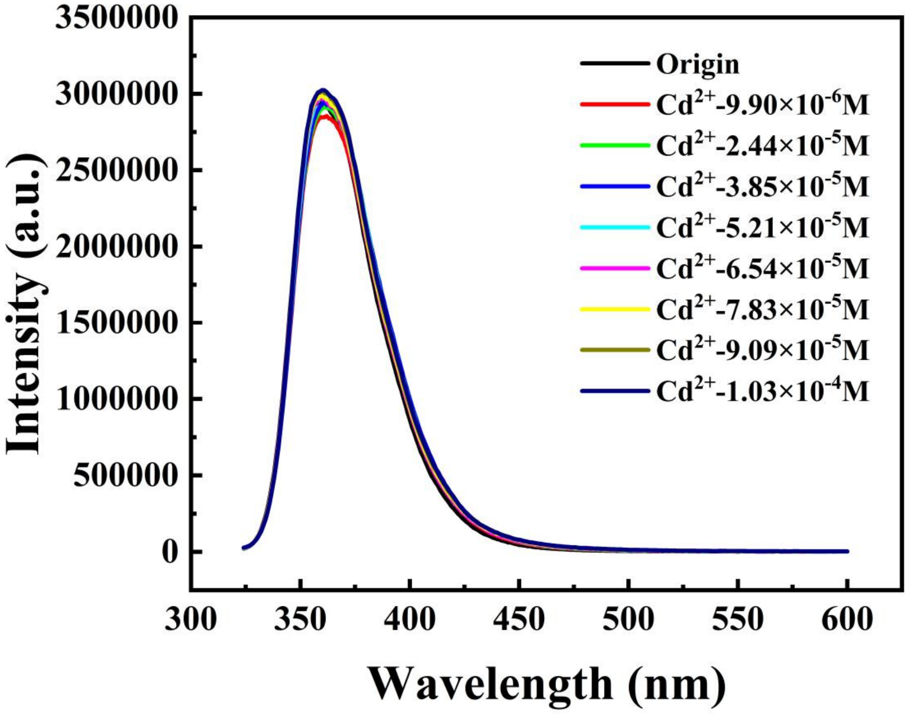

Figure S3. The PL spectrum of HOF-TCBP with different amounts of Cd^{2+}.

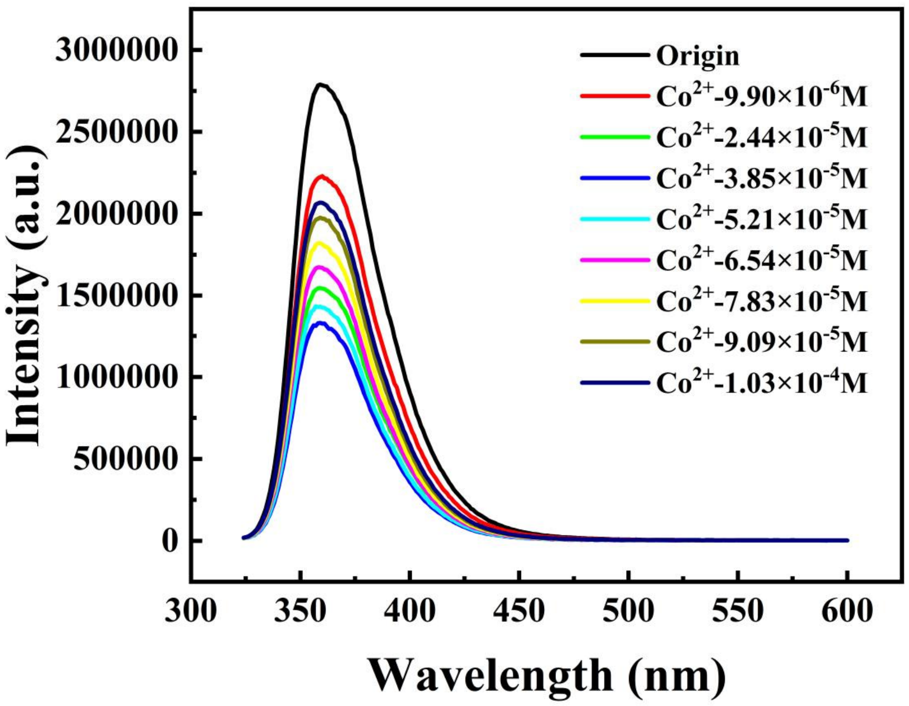

Figure S4. The PL spectrum of HOF-TCBP with different amounts of Co^{2+}.

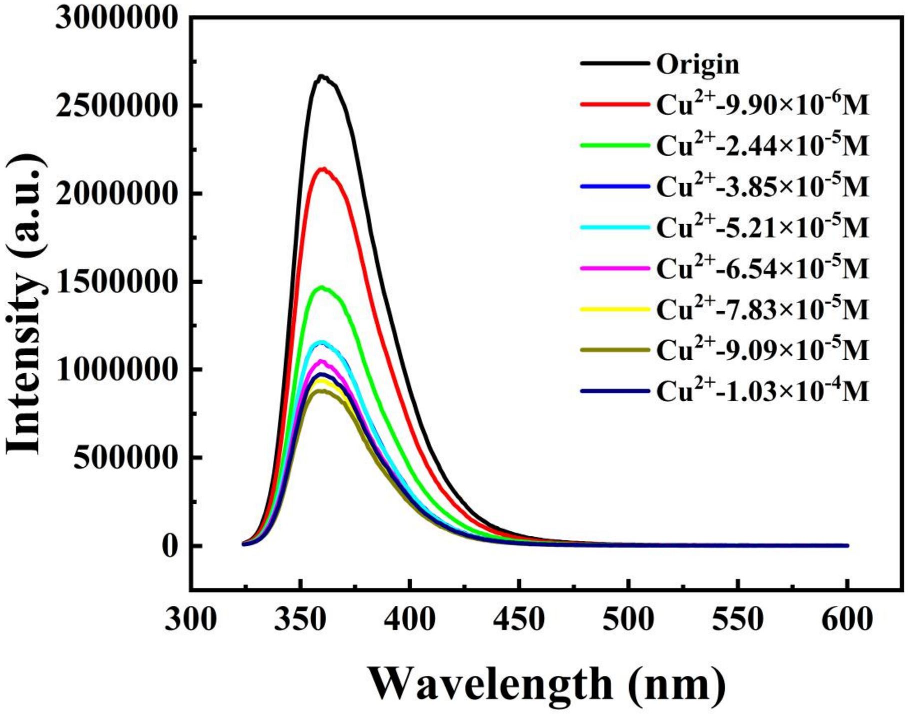

Figure S5. The PL spectrum of HOF-TCBP with different amounts of Cu^{2+}.

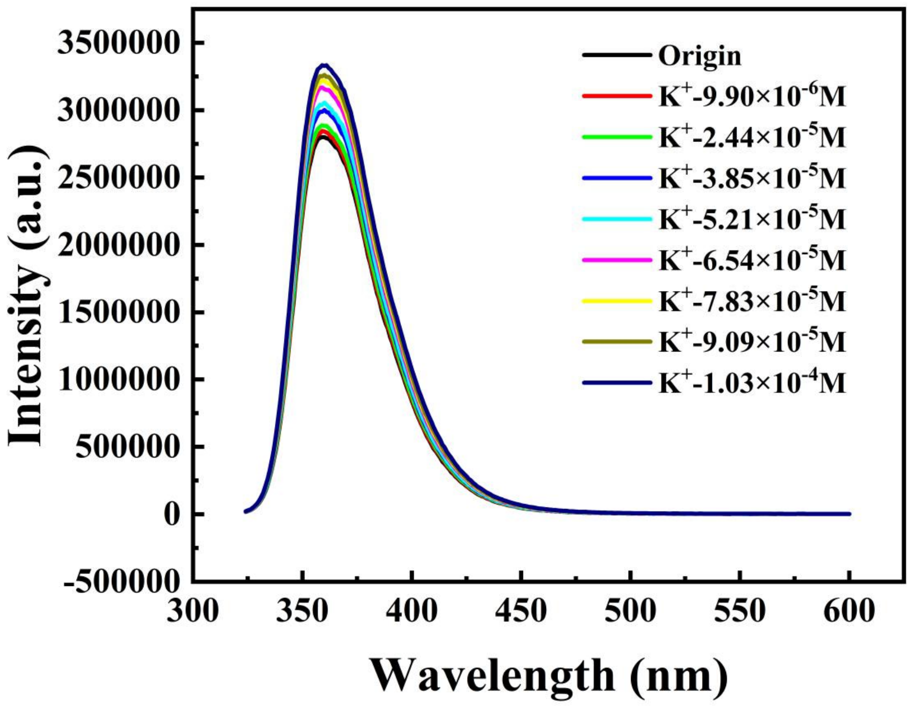

Figure S6. The PL spectrum of HOF-TCBP with different amounts of K^+.

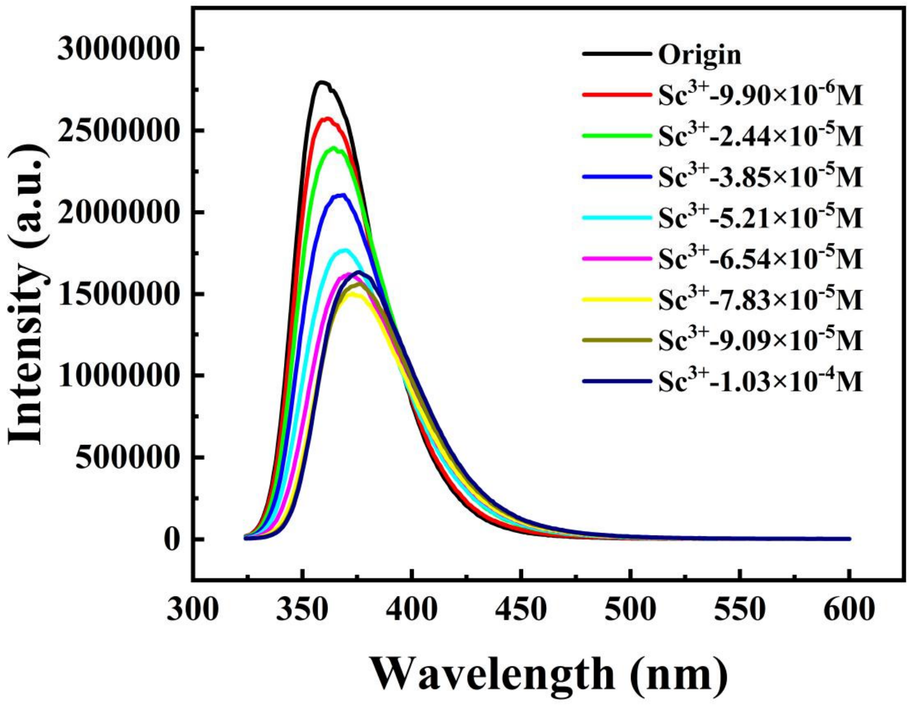

Figure S7. The PL spectrum of HOF-TCBP with different amounts of Sc^{3+}.

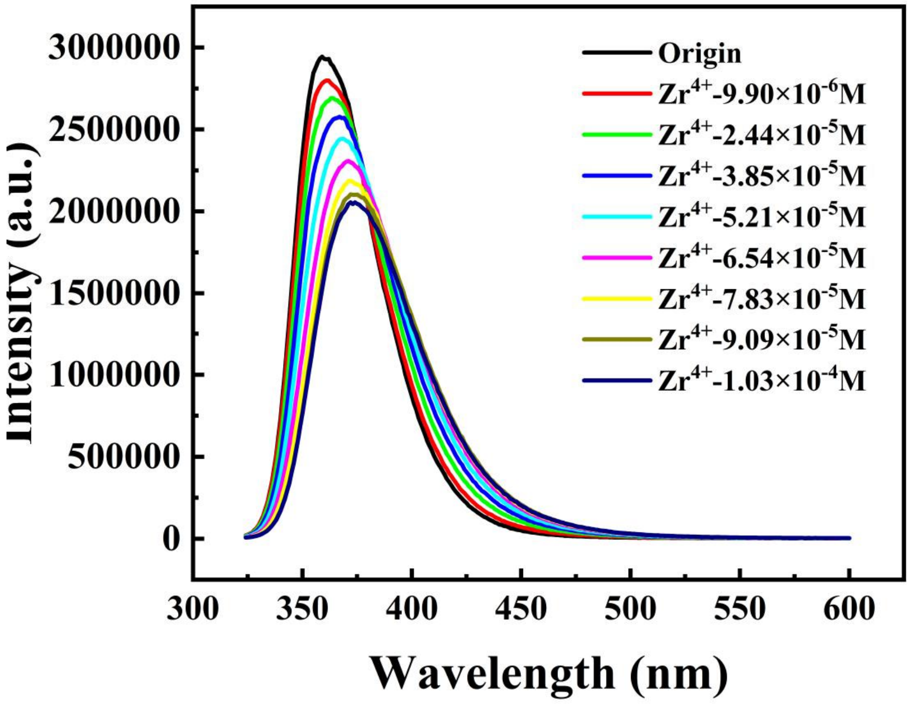

Figure S8. The PL spectrum of HOF-TCBP with different amounts of Zr^{4+}.

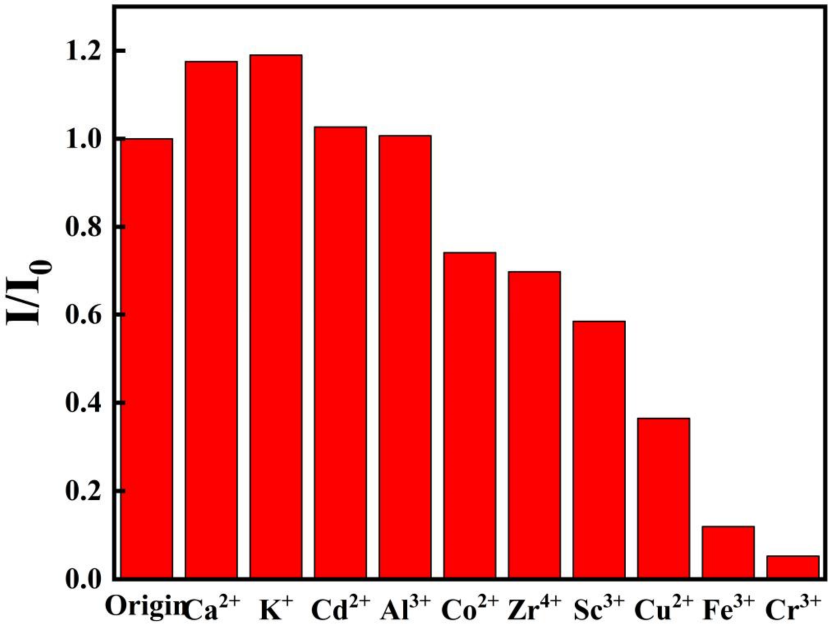

Figure S9. The 359 nm emission intensity of HOF-TCBP ethanol solution with 1.0×10^{-3} M different

metal ion.

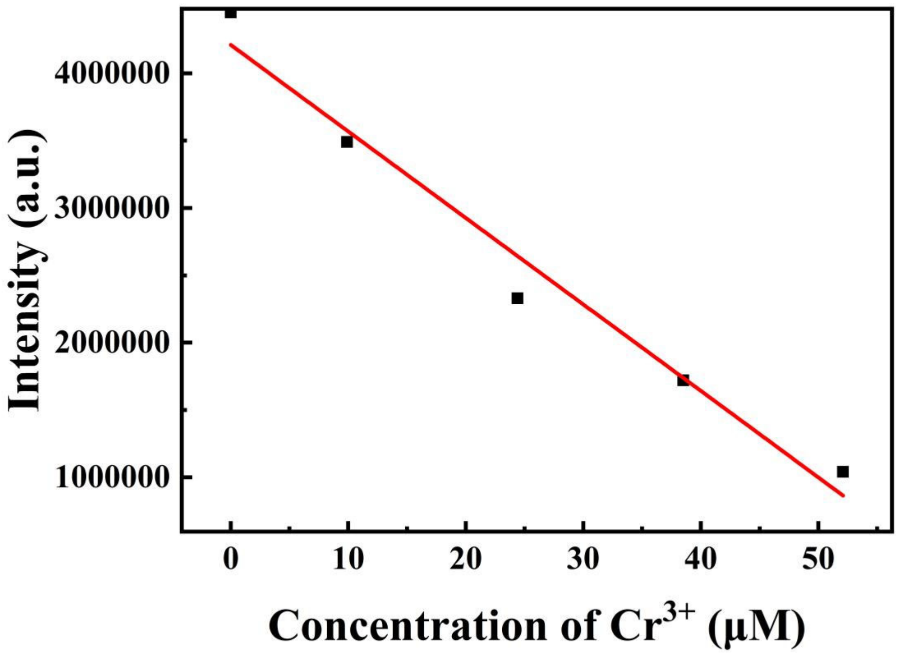

Figure S10. The fit curve of HOF-TCBP emission intensity at different Cr^{3+} concentrations.

Linear Equation: Y=-64230.019X+4211143.876

$S=6.4230019\times10^{10}\ M^{-1}$

$I_a=\frac{4229918+4218369+4221680+4194120+4191930+4198920+4197870+4196500+4199690+4184230}{10}$

=4203322.7

$S_b=\sqrt{\frac{\sum(I_0-I_a)^2}{N-1}}$=14751.90207   (N=8)

$LOD=\frac{3\times S_b}{S}=\frac{3\times14751.90207}{6.4230019\times10^{10}}$=0.689 μM

S is the slope of the calibration curve, S_b is the standard deviation of the blank group, I_0 is the fluorescence intensity of the HOF solution, and I_a is the average of I_0.

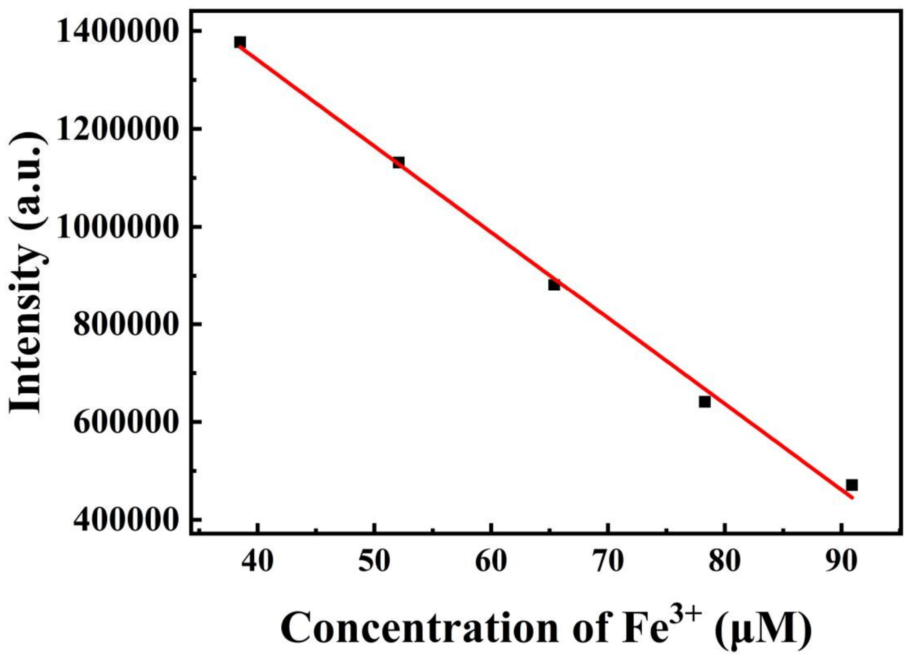

Figure S11. The fit curve of HOF-TCBP emission intensity at different Fe^{3+} concentrations.

Linear Equation: Y=-17588.733X+2044301.391

$S=1.7588733\times10^{10}\ M^{-1}$

$I_a=\frac{4229918+4218369+4221680+4194120+4191930+4198920+4197870+4196500+4199690+4184230}{10}$

=4203322.7

$S_b=\sqrt{\frac{\sum(I_0-I_a)^2}{N-1}}$=14751.90207 (N=8)

$LOD=\frac{3\times S_b}{S}=\frac{3\times14751.90207}{1.7588733\times10^{10}}$=2.516 μM

S is the slope of the calibration curve, S_b is the standard deviation of the blank group, I_0 is the fluorescence intensity of the HOF solution, and I_a is the average of I_0.

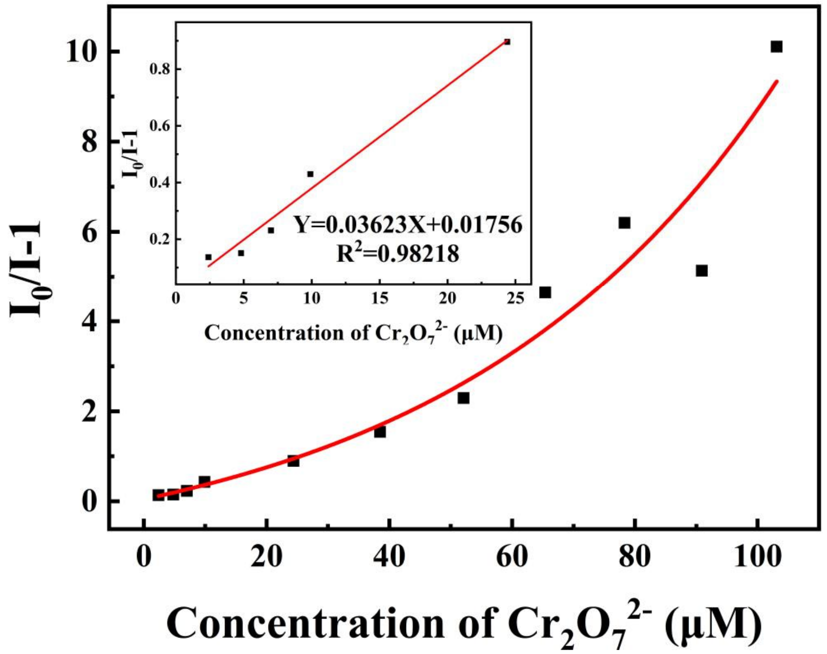

Figure S12. S–V plots of $Cr_2O_7^{2-}$; inset: the above figures are the linear fitting curves at low concentrations.

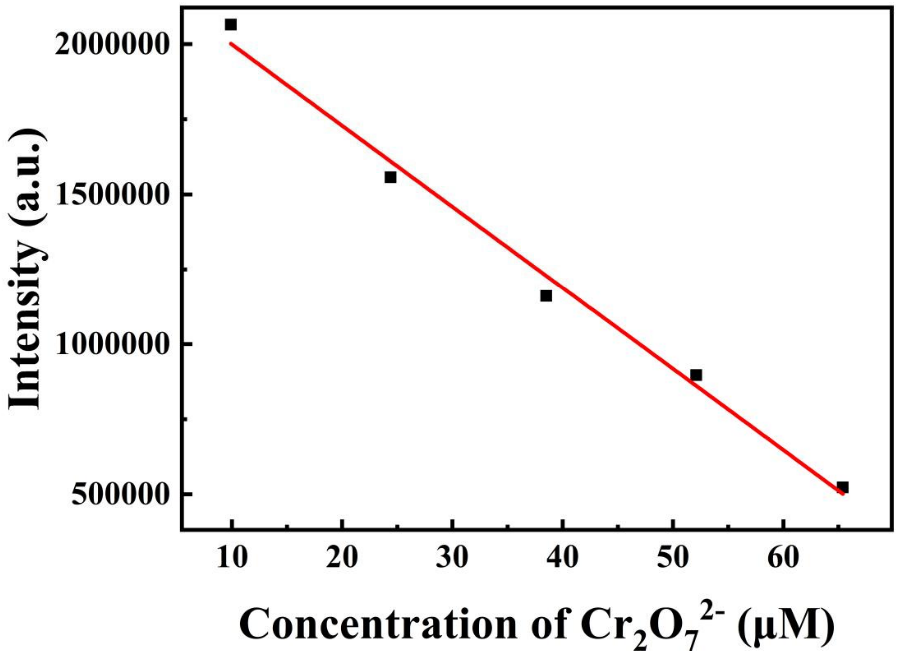

Figure S13. The fit curve of HOF-TCBP emission intensity at different $Cr_2O_7^{2-}$ concentrations.

Linear Equation: Y=-27023.959X+2269090.727

S=2.7023959×10^{10} M^{-1}

$$I_a = \frac{4229918+4218369+4221680+4194120+4191930+4198920+4197870+4196500+4199690+4184230}{10}$$

=4203322.7

$$S_b = \sqrt{\frac{\sum(I_0 - I_a)^2}{N-1}} = 14751.90207 \qquad (N=8)$$

$$LOD = \frac{3 \times S_b}{S} = \frac{3 \times 14751.90207}{2.7023959 \times 10^{10}} = 1.638\ \mu M$$

S is the slope of the calibration curve, S_b is the standard deviation of the blank group, I_0 is the fluorescence intensity of the HOF solution, and I_a is the average of I_0.

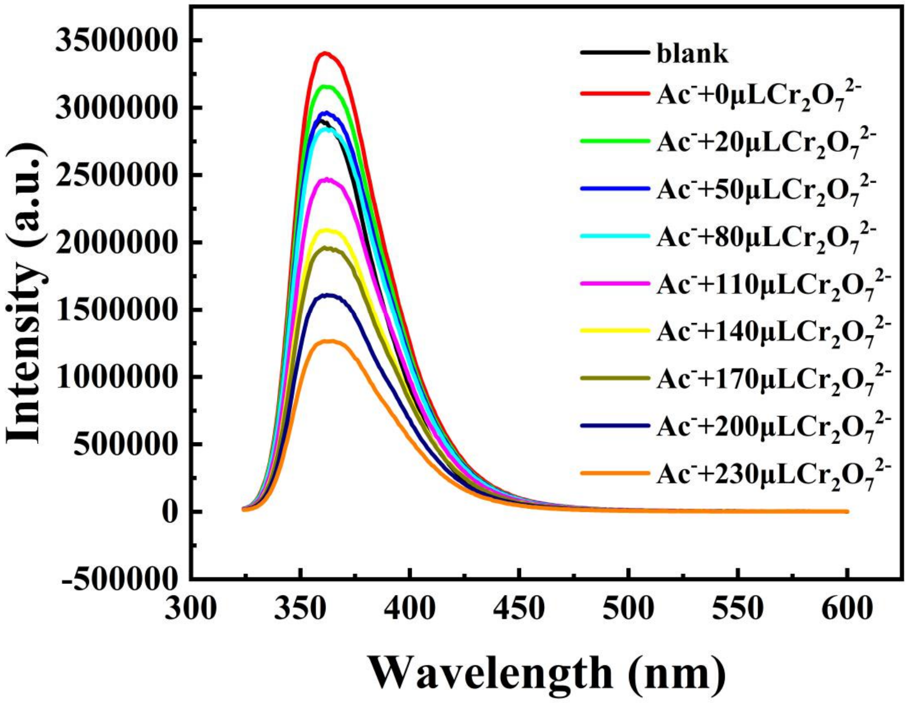

Figure S14. The change of fluorescence intensity of HOF-TCBP after adding Ac^- (230 μL, 10^{-3} M) and different contents of $Cr_2O_7^{2-}$ (10^{-3} M).

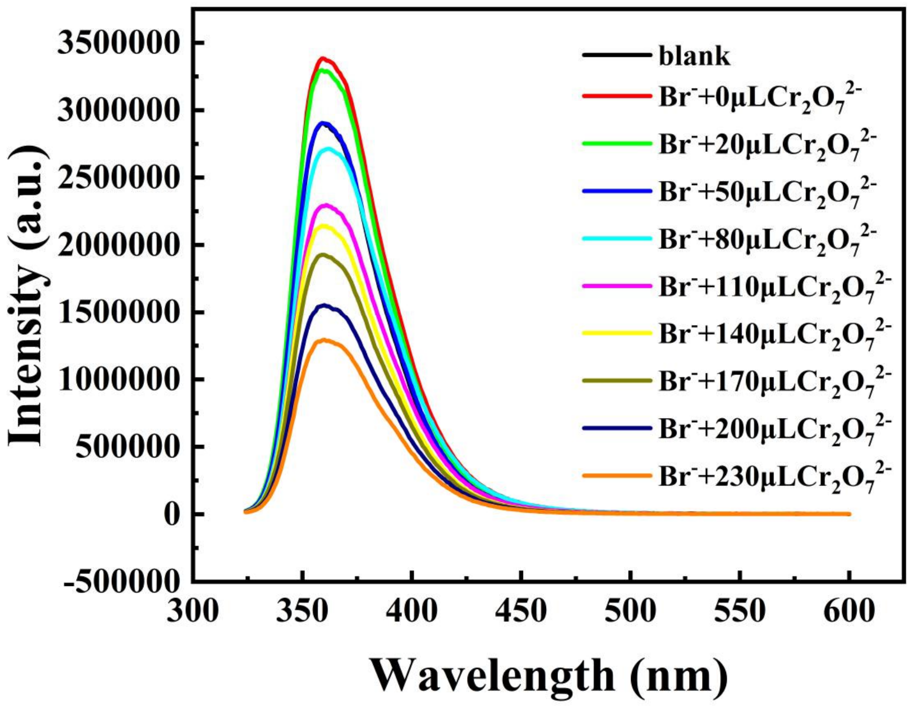

Figure S15. The change of fluorescence intensity of HOF-TCBP after adding Br^- (230 μL, 10^{-3} M) and different contents of $Cr_2O_7^{2-}$ (10^{-3} M).

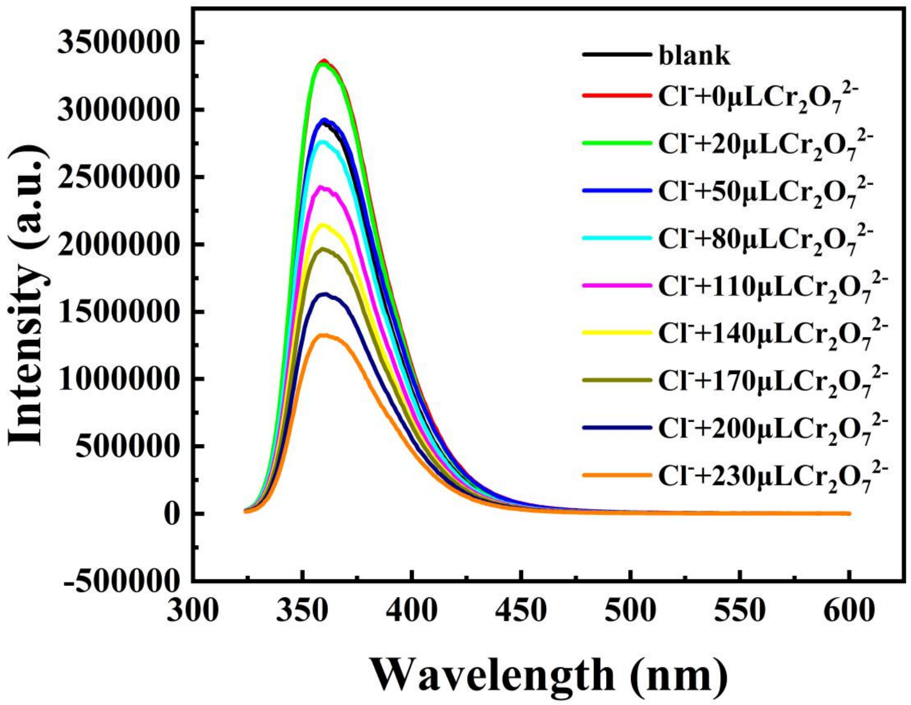

Figure S16. The change of fluorescence intensity of HOF-TCBP after adding Cl^- (230 μL, 10^{-3} M) and

different contents of $Cr_2O_7^{2-}$ (10^{-3} M).

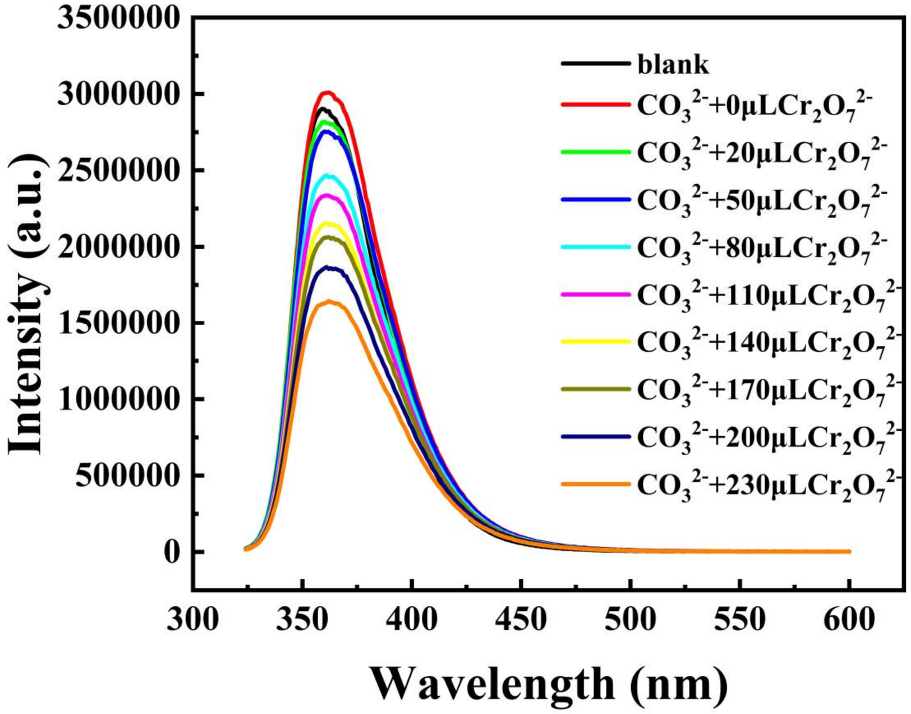

Figure S17. The change of fluorescence intensity of HOF-TCBP after adding CO_3^{2-} (230 μL, 10^{-3} M) and different contents of $Cr_2O_7^{2-}$ (10^{-3} M).

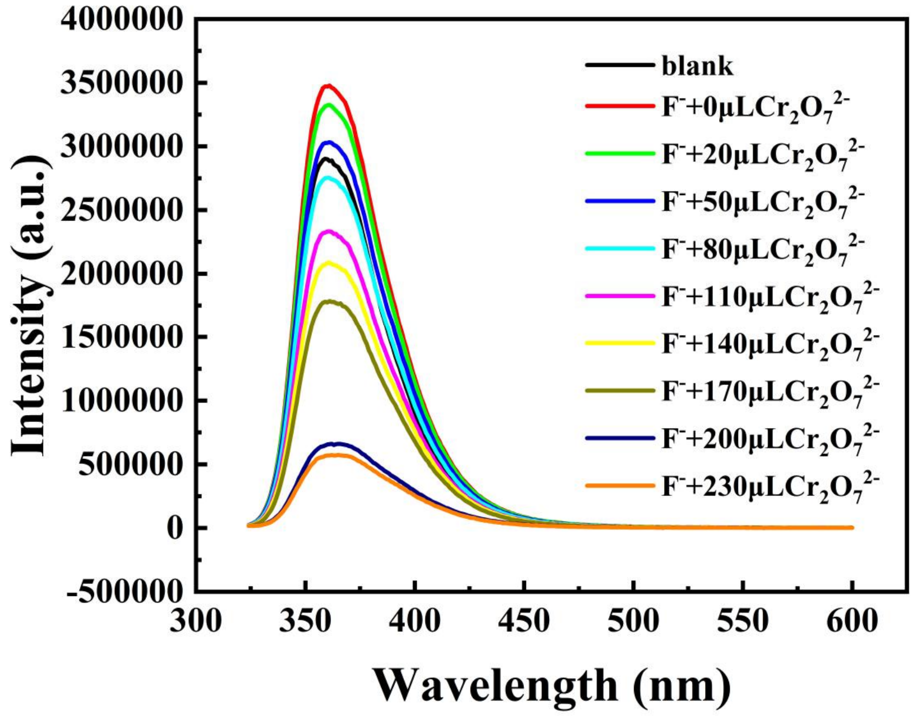

Figure S18. The change of fluorescence intensity of HOF-TCBP after adding F^- (230 μL, 10^{-3} M) and different contents of $Cr_2O_7^{2-}$ (10^{-3} M).

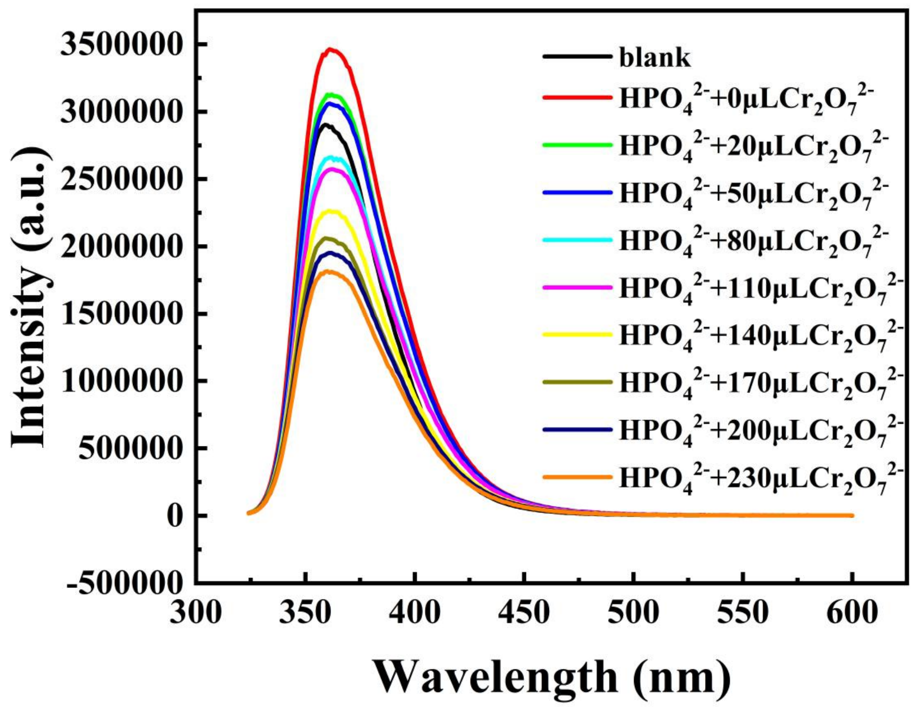

Figure S19. The change of fluorescence intensity of HOF-TCBP after adding HPO_4^{2-} (230 μL, 10^{-3}

M) and different contents of $Cr_2O_7^{2-}$ (10^{-3} M).

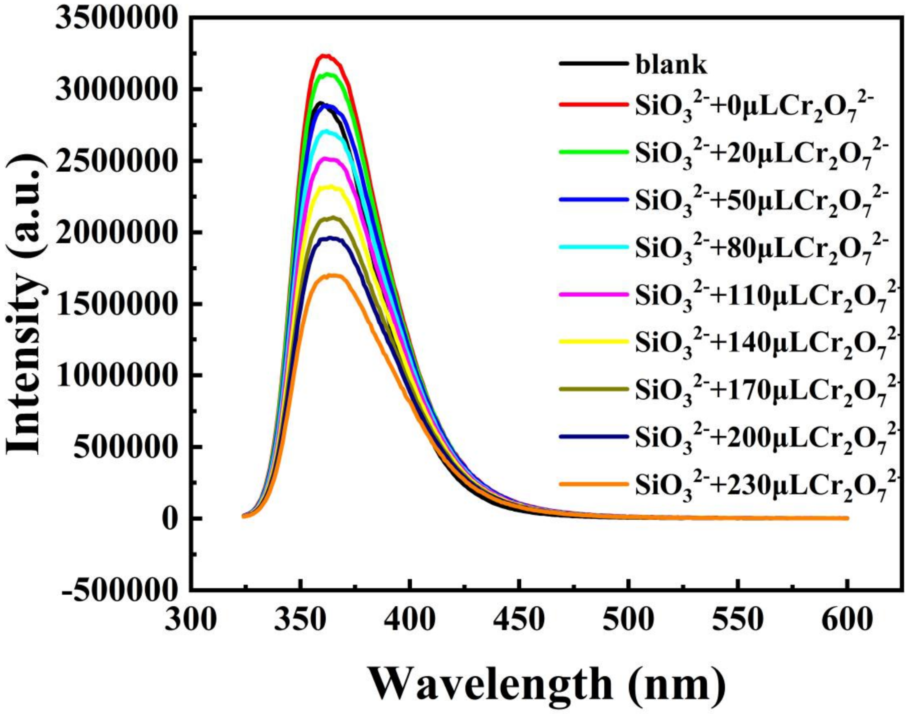

Figure S20. The change of fluorescence intensity of HOF-TCBP after adding SiO_3^{2-} (230 μL, 10^{-3} M) and different contents of $Cr_2O_7^{2-}$ (10^{-3} M).

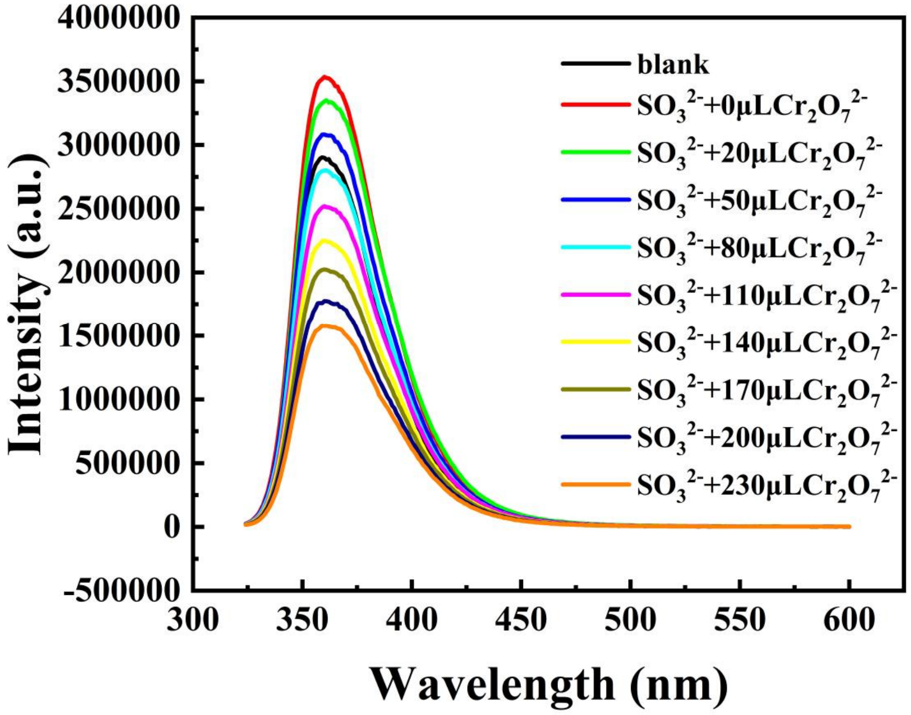

Figure S21. The change of fluorescence intensity of HOF-TCBP after adding SO_3^{2-} (230 μL, 10^{-3} M) and different contents of $Cr_2O_7^{2-}$ (10^{-3} M).

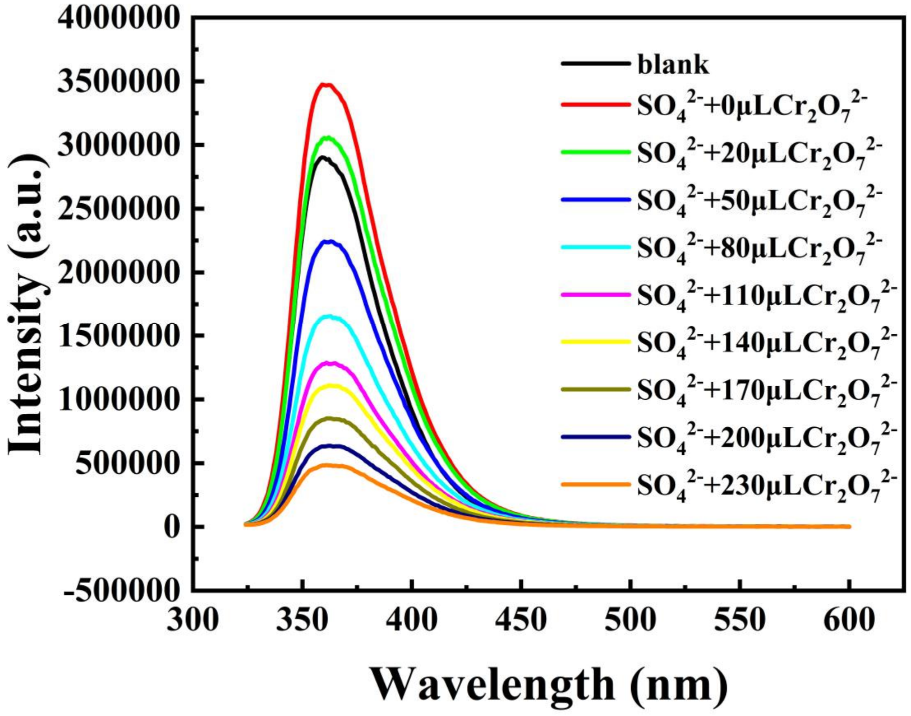

Figure S22. The change of fluorescence intensity of HOF-TCBP after adding SO_4^{2-} (230 μL, 10^{-3} M) and

different contents of $Cr_2O_7^{2-}$ (10^{-3} M).

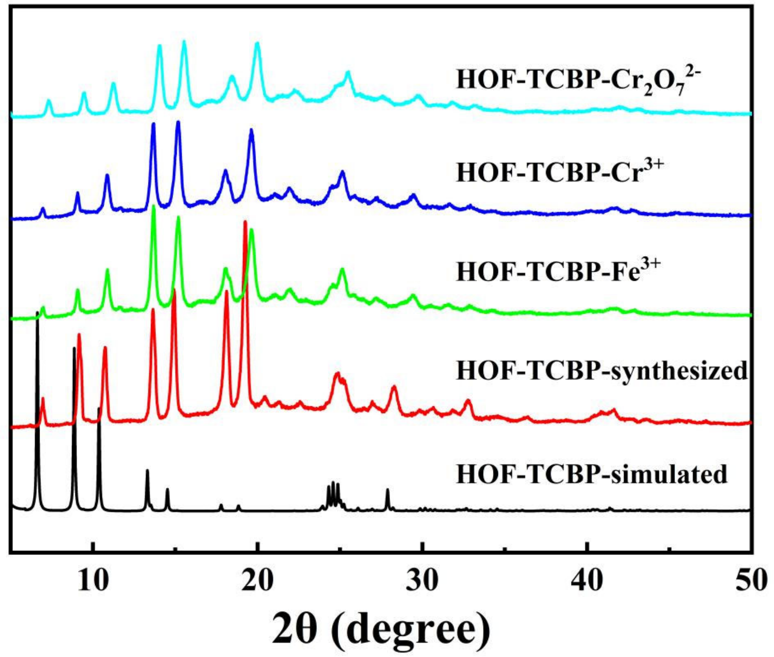

Figure S23. PXRD patterns of HOF-TCBP and HOF-TCBP immersed in Cr^{3+}, Fe^{3+} and $Cr_2O_7^{2-}$ aqueous solutions (10^{-3} M).

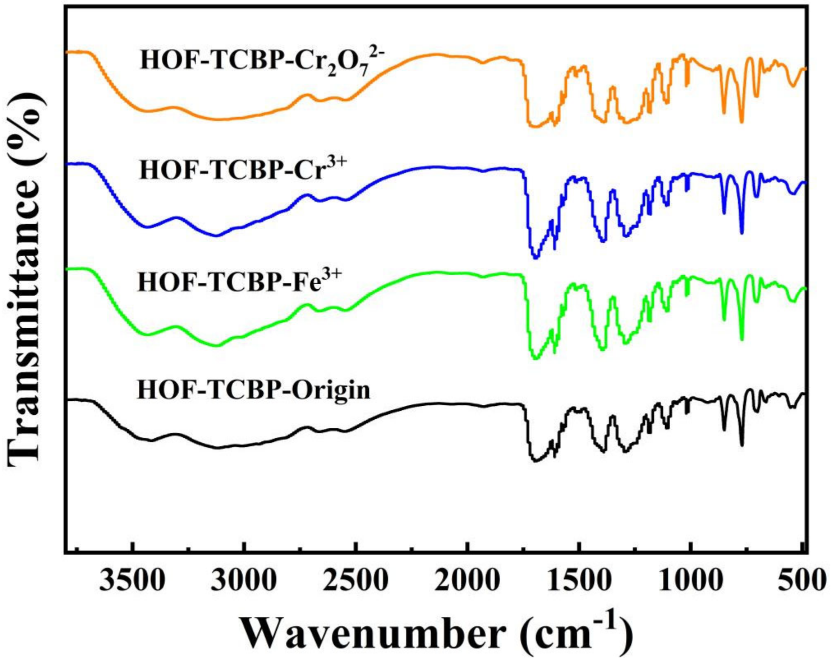

Figure S24. FT-IR spectra of HOF-TCBP and HOF-TCBP immersed in Cr^{3+}, Fe^{3+} and $Cr_2O_7^{2-}$ aqueous solutions (10^{-3} M).

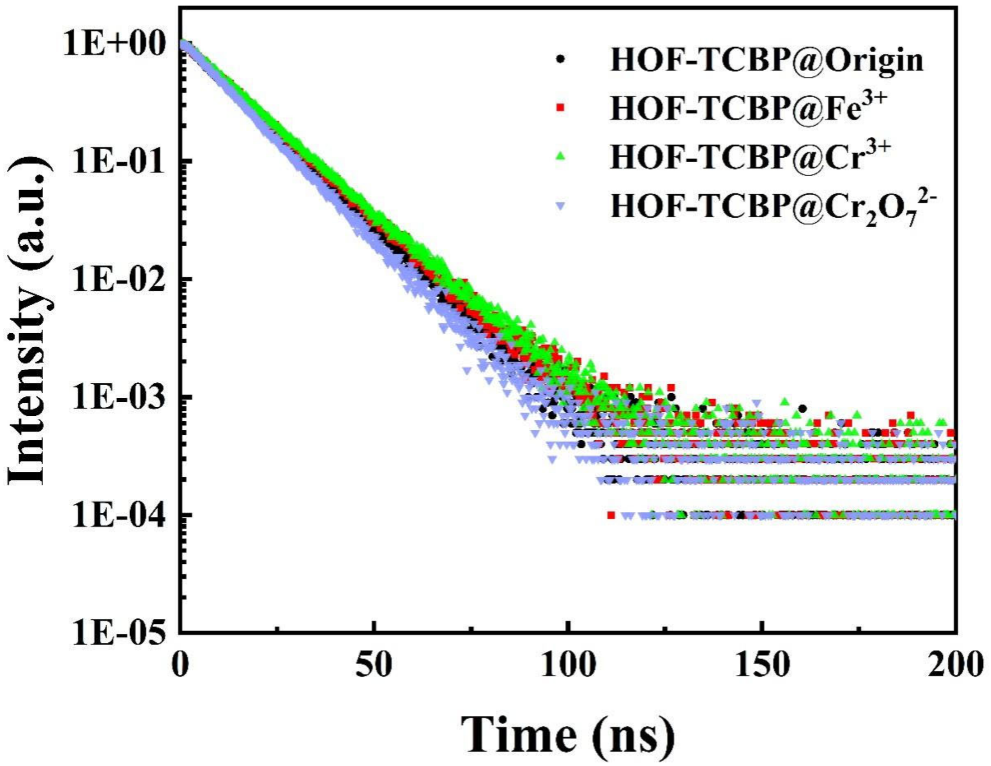

Figure S25. HOF-TCBP fluorescence lifetimes monitored at 359 nm with and without ion addition.

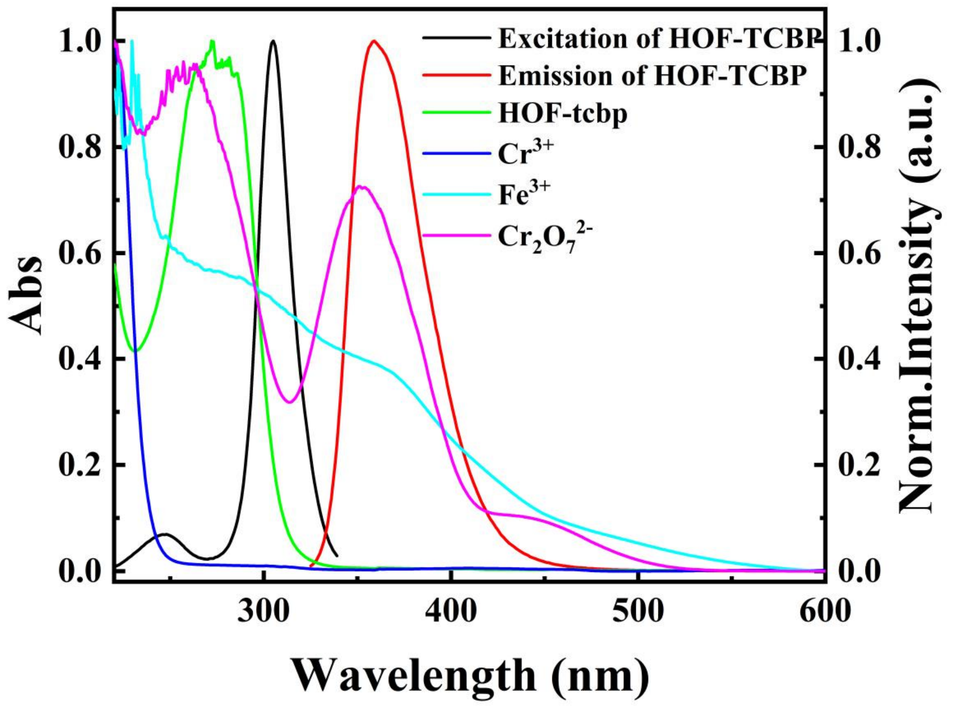

Figure S26. Absorption spectra of ions and excitation and emission spectra of HOF-TCBP.

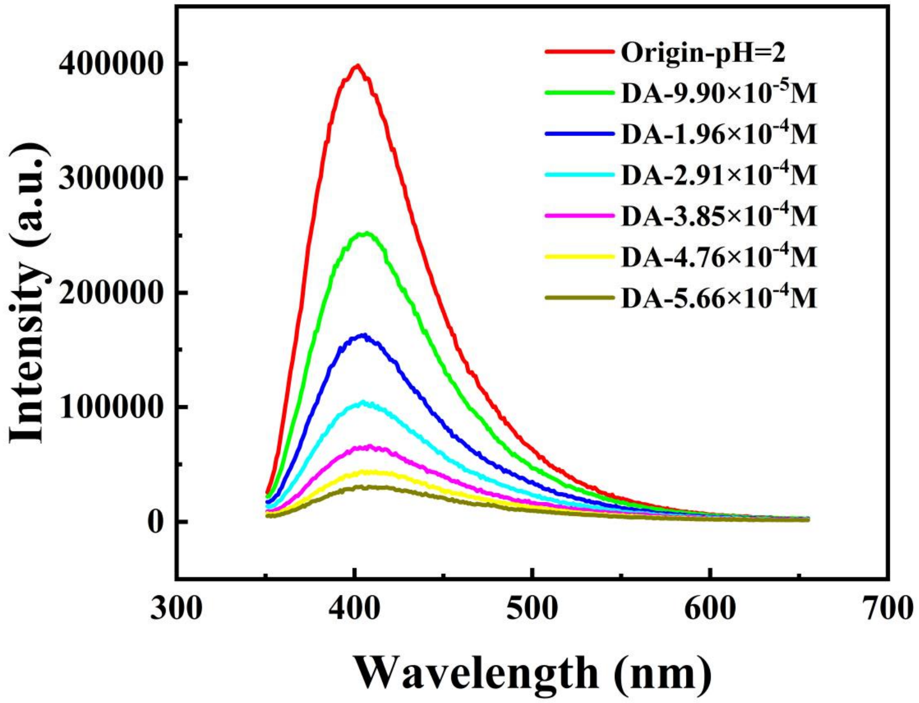

Figure S27. PL spectra of HOF-TCBP with different DA contents in pH=2 buffer solution.

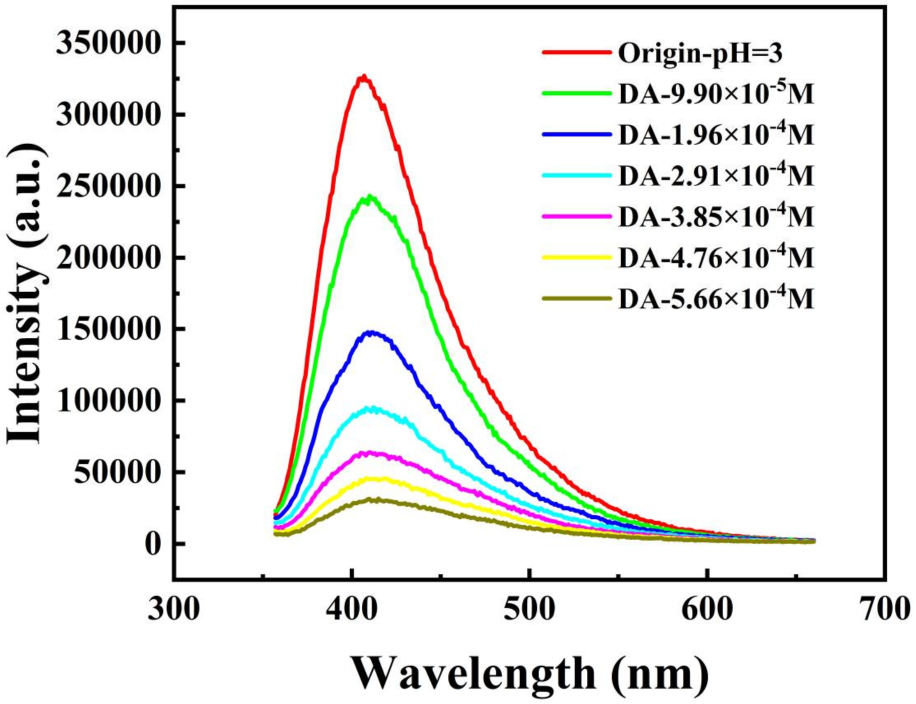

Figure S28. PL spectra of HOF-TCBP with different DA contents in pH = 3 buffer solution.

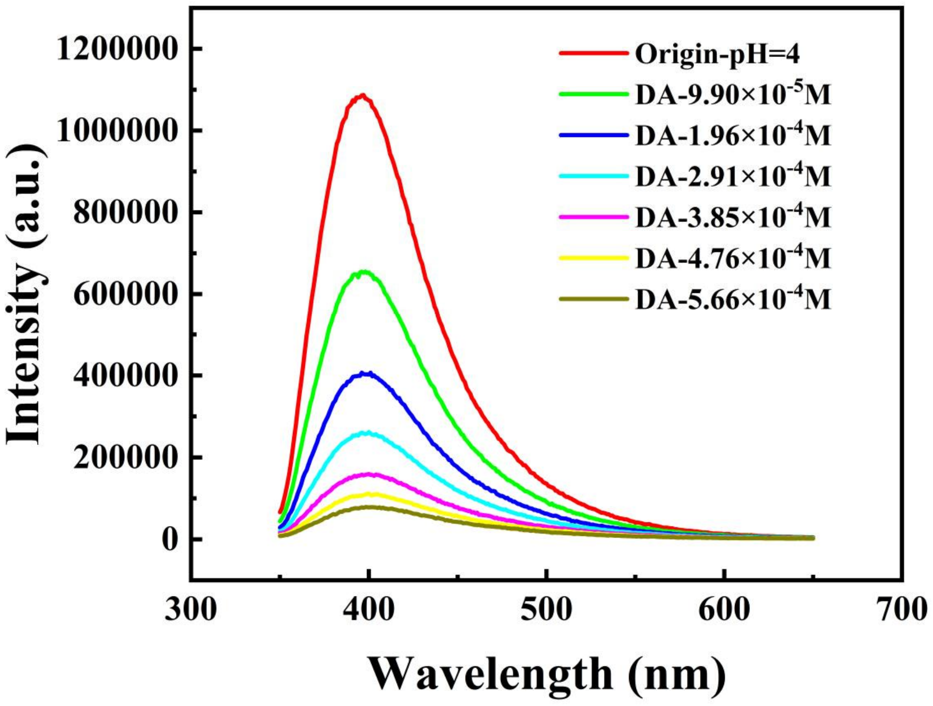

Figure S29. PL spectra of HOF-TCBP with different DA contents in pH = 4 buffer solution.

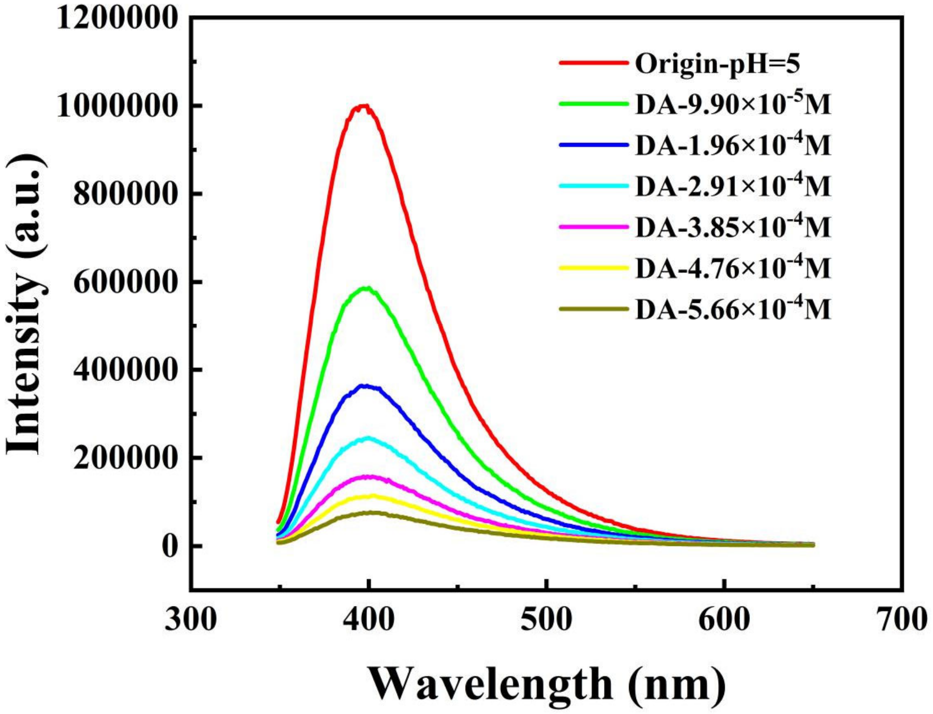

Figure S30. PL spectra of HOF-TCBP with different DA contents in pH = 5 buffer solution.

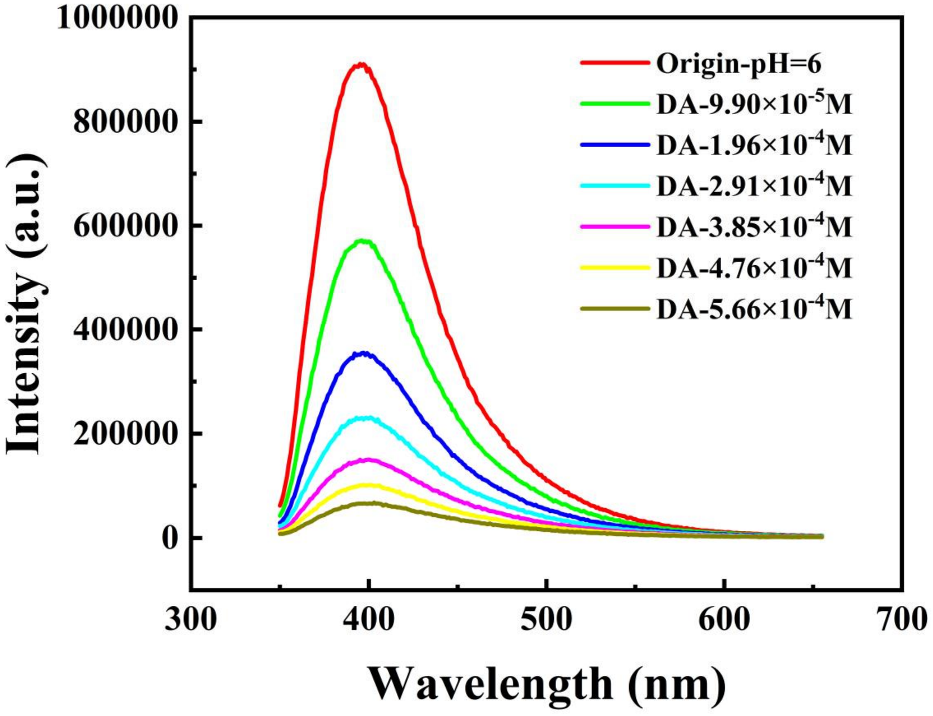

Figure S31. PL spectra of HOF-TCBP with different DA contents in pH = 6 buffer solution.

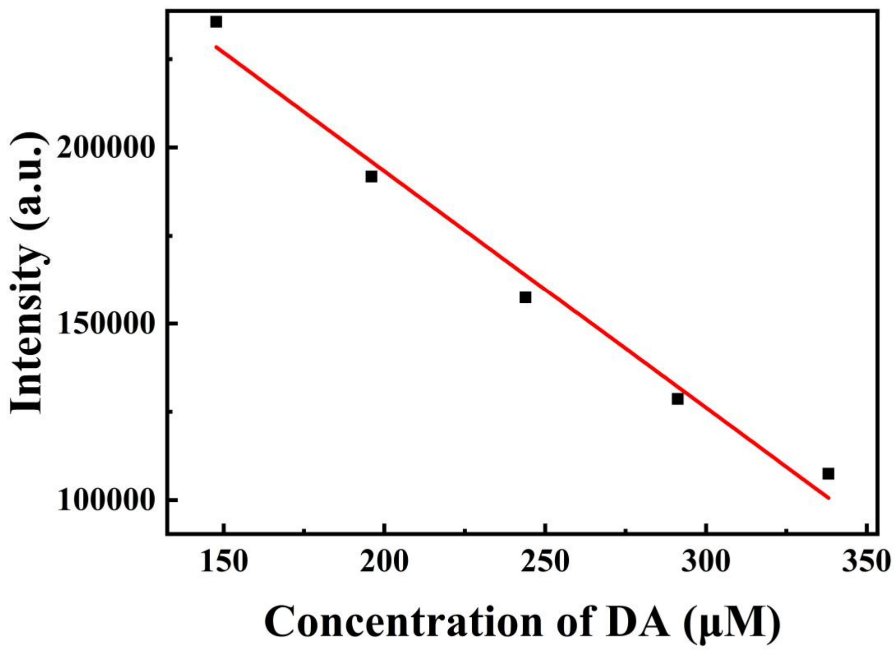

Figure S32. The fit curve of HOF-TCBP emission intensity at different DA concentrations.

Linear Equation: Y=-671.37516X+327573.67977

$S=6.7137516\times10^{8}$ M^{-1}

$I_a=\frac{573293.7+589806.6+583444.1+583242.7+571631.2+566931.1+581790.5+569683.1+565883.4+580910.8}{10}$

=576661.72

$S_b=\sqrt{\frac{\sum(I_0-I_a)^2}{N-1}}$=8183.077976 (N=8)

$LOD=\frac{3\times S_b}{S}=\frac{3\times8183.077976}{6.7137516\times10^{8}}$=36.57 μM

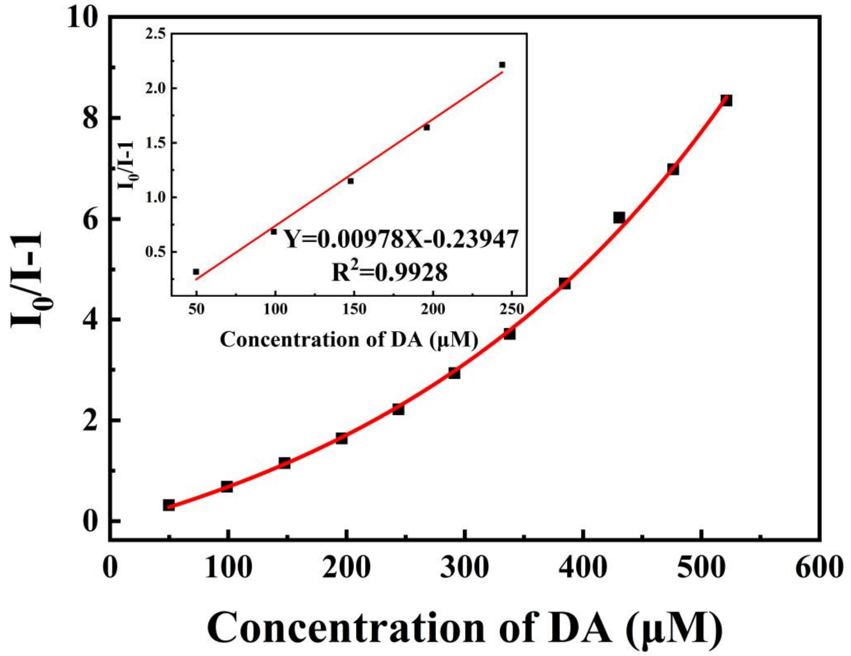

Figure S33. S–V plots of DA; inset: the above figures are the linear fitting curves at low concentrations.

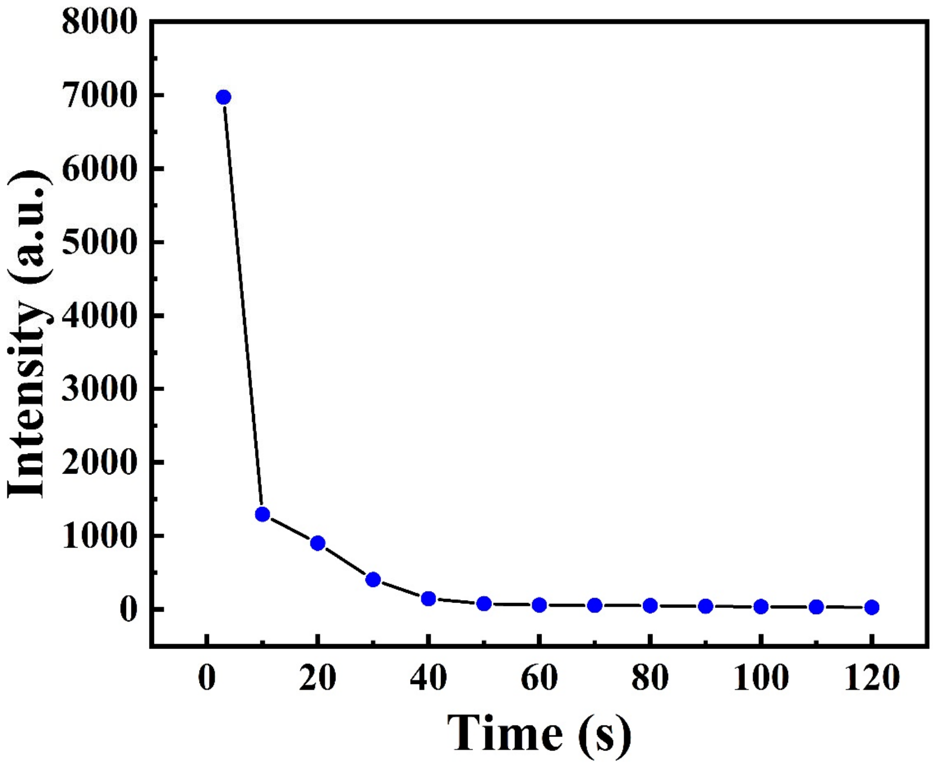

Figure S34. After adding a certain concentration of DA, the luminescence intensity of HOF-TCBP

suspension at 397 nm changes with time.

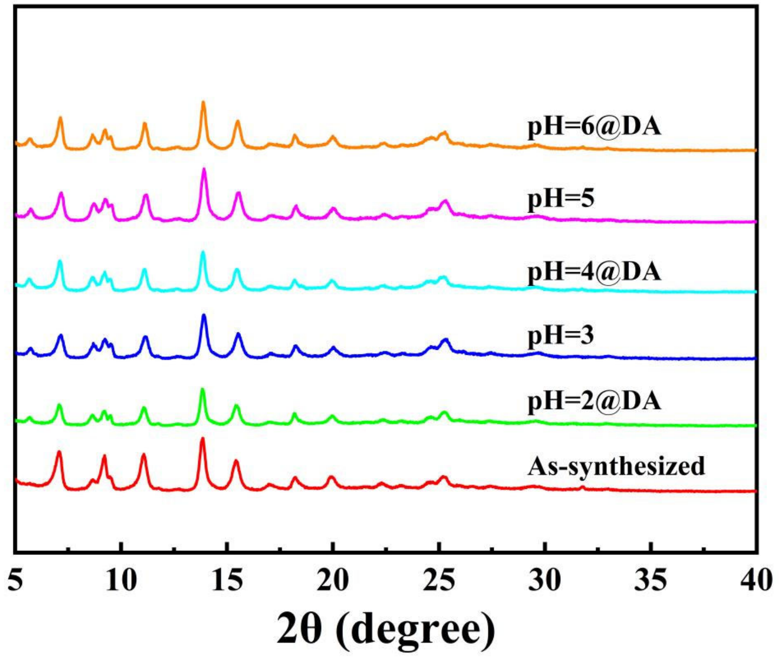

Figure S35. PXRD patterns of HOF-TCBP synthesized samples and immersed in pH = 2 - 6 buffer solution and DA solution (10^{-2} M).

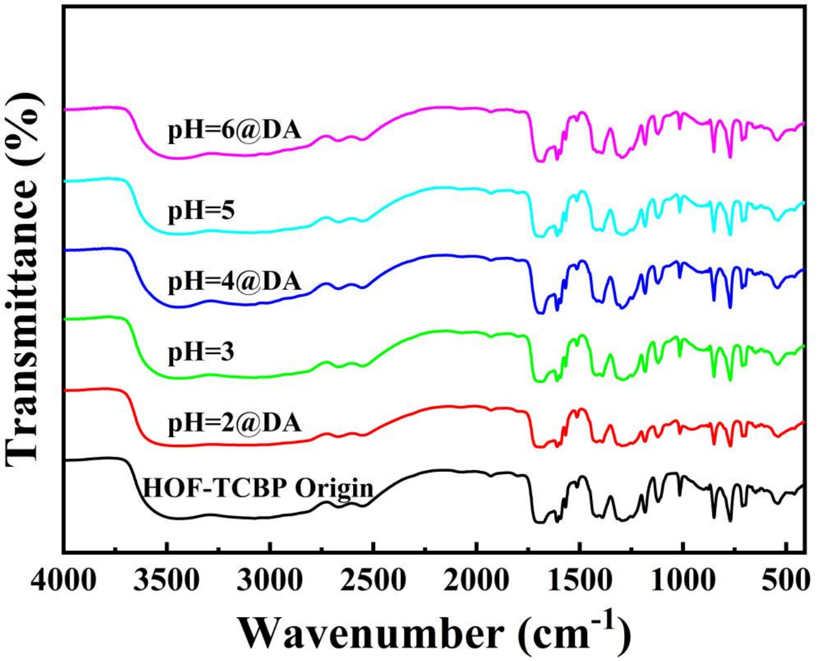

Figure S36. FT-IR spectra of HOF-TCBP synthesized samples and immersed in pH = 2 - 6 buffer solution and DA solution (10^{-2} M).

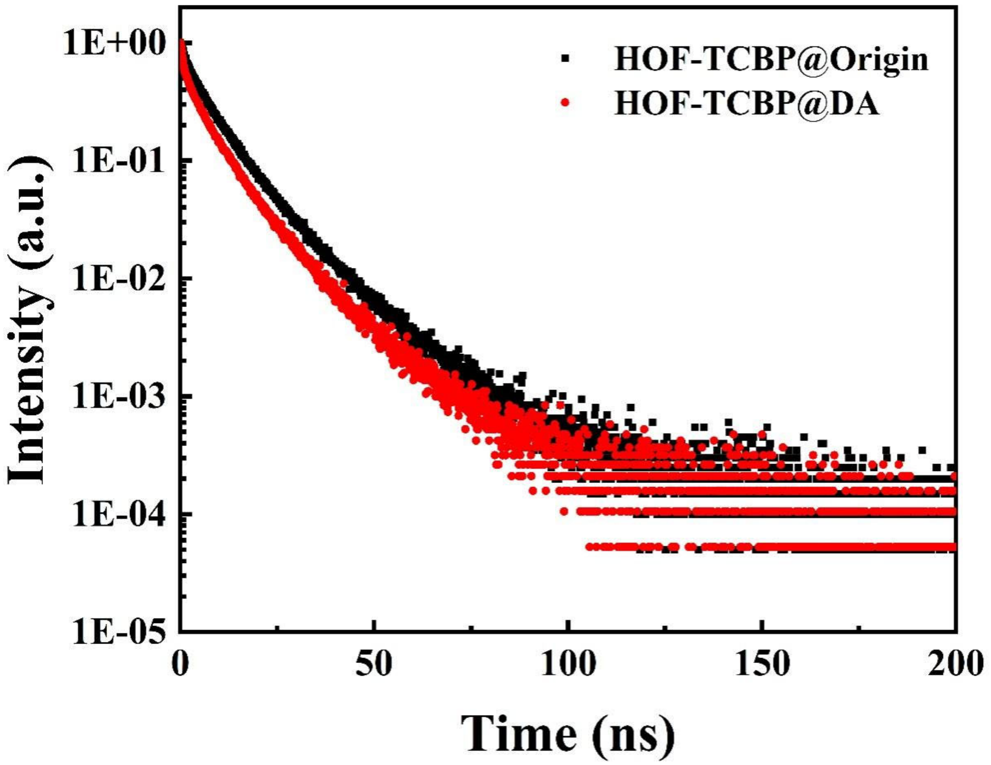

Figure S37. HOF-TCBP fluorescence lifetime monitored at 397 nm without and with DA addition.

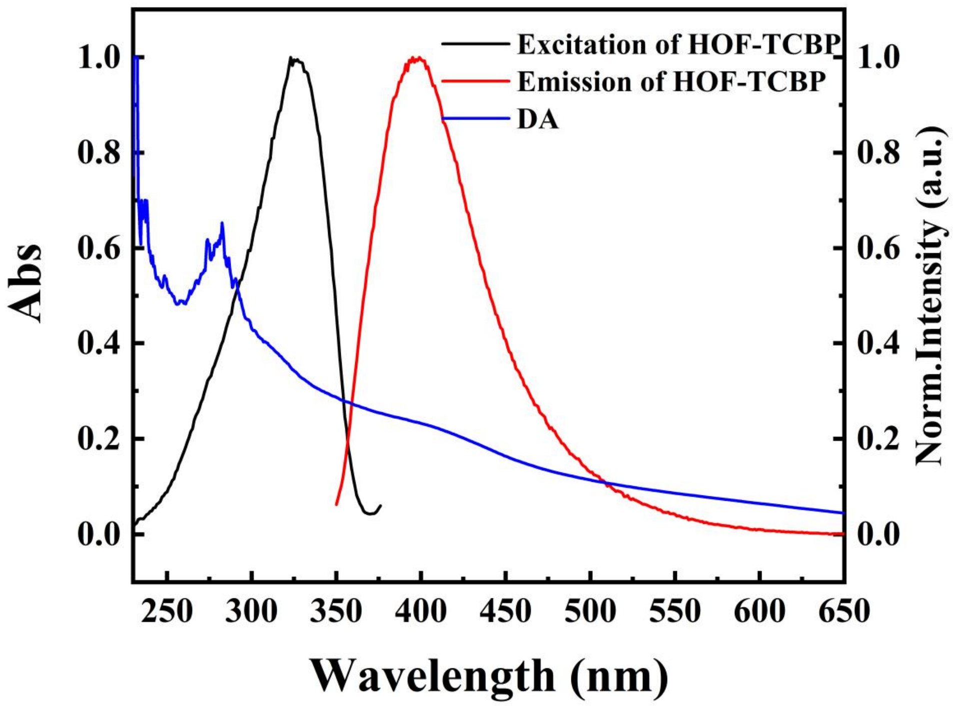

Figure S38. Absorption spectra of DA and excitation and emission spectra of HOF-TCBP.

Table S1. Comparison of luminescent materials for detecting Fe^{3+} ion.

Material	Solvent	$K_{sv}(M^{-1})$	Detection limit(mM)	Ref.
Ag(bpy)(IPA-OH)	H_2O	-	1.2	[1]
Ag(bpy)(IPA-2,6-NDC)	H_2O	-	1.8	[1]
PAF-5CF	EtOH	11865	38	[2]
FJI-C8	DMF	8245	23.3	[3]
$[Eu2(ppda)_2(npdc)(H_2O)_2]n$	H_2O	1.64×10^5	16.6	[4]
$\{[Cd_3(BPDPE)(BDC)_3\cdot(DMF)_2]2DMF\cdot2H_2O\}n$	DMF	8.57×10^3	10	[5]
$[Cd_2(TFBA)(HCOO)$ $(bpe)H_2O]n$	H_2O	1.03×10^6	2.49	[6]
$[Tb(TBOT)(H_2O)](H_2O)_4(DMF)(NMP)_{0.5}$	H_2O	5.51×10^3	130	[7]
$[Cd(Hcbic)]_n$	H_2O	1.8×10^5	31	[8]
$[Ln_2L(1,3\text{-}bdc)_3]\cdot5H_2O$	H_2O	1.2×10^4	23	[9]
BUT-14	H_2O	2.2×10^3	3.8	[10]
BUT-15	H_2O	1.7×10^4	0.3	[10]
**HOF-TCBP**	**Ethanol**	$\mathbf{3.01\times10^4}$	**2.516**	**This work**

Table S2. Comparison of luminescent materials for detecting Cr^{3+} ion.

Material	Solvent	$K_{sv}(M^{-1})$	Detection limit(mM)	Ref.
$[Eu_2(ppda)_2(npdc)(H_2O)]\cdot H_2O$	H_2O	1.98×10^6	61.7	[3]
$[Zn(L)(H_2O)]\cdot H_2O$	H_2O	2.03×10^4	2.44	[11]
$\{[Zn_2(\mu_3\text{-}OH)(cpta)(4,4\text{ -}bipy)]\cdot H_2O\}n$	H_2O	9.47×103	5.55	[12]
$[Zn_2(TPOM)(BDC)_2]\cdot4H_2O$	DMF	-	4.9	[13]
$[Eu_2(tpbpc)_4\cdot CO_3\cdot H_2O]\cdot$ DMF·solvent	H_2O	5.14×10^2	3.64	[14]
$Zn_3(bpdc)_2(pdc)(DMF)\cdot6DMF$	H_2O	3.87×10^3	25.1	[15]
[Zn (tbda)]n	H_2O	2.68×10^3	180	[16]
YF_3: Eu^{3+} nanoparticles	H_2O	6.458×10^3	1.88	[17]
PVP@Gd_2O_3:Eu^{3+} NPs	H_2O	-	2.1	[18]
S/N-CQDs	H_2O	-	6.0	[19]
$\{[Cd_2(adc)_2(4\text{-}nvp)_6]\cdot MeOH\cdot H_2O\}n$	CH_3CN /H_2O	-	0.31	[20]
**HOF-TCBP**	**Ethanol**	$\mathbf{8.5\times10^4}$	**0.689**	**This work**

Table S3. Comparison of luminescent materials for detecting $Cr_2O_7^{2-}$ ion.

Material	Solvent	$K_{sv}(M^{-1})$	Detection limit(mM)	Ref.
Zn-MOF-**1**	H_2O	2.07×10^4	3.53	[11]
$[Zn_2(TPOM)(BDC)_2]\cdot4H_2O$	DMF	7.59×10^3	3.9	[13]

Eu_4L_3	DMF	1.526×10^3	10	[21]
1-Eu	Ethanol	-	22	[22]
[Eu_2(tpbpc)$_4$·CO_3·H_2O]·DMF·solvent	H_2O	1.04×10^4	4.95	[14]
[Tb(TATAB)(H_2O)$_2$]·NMP·H_2O}n	H_2O	-	1.0	[23]
[Zn_2(4,4′-nba)$_2$(1,4-bib)$_2$]n	H_2O	6.7×10^3	3.8	[24]
[Zn(IPA)(3-PN)]n	H_2O	1.37×10^3	12	[25]
[Cd(IPA)(3-PN)]n	H_2O	2.91×10^3	2.26	[25]
**HOF-TCBP**	**Ethanol**	**3.62×10^4**	**1.638**	**This work**

References:

[1] Van Nguyen, C. ; Matsagar, B.M.; Ahamad, T.; Alshehri, S.M.; Chiang, W.-H.; Wu,K.C.W. Unraveling the highly selective nature of silver-based metal–organic complexes for the detection of metal ions: the synergistic effect of dicarboxylic acid linkers. *J. Mater. Chem. C* **2020**, *8*, 5051–5057.

[2] Ma, T.; Zhao, X.; Matsuo, Y.; Song, J.; Zhao, R.; Faheem, M.; Chen, M.; Zhang, Y.; Tian, Y.; Zhu, G. Fluorescein-based fluorescent porous aromatic framework for Fe3+ detection with high sensitivity. *J. Mater. Chem. C* **2019**, *7*, 2327–2332.

[3] Zhan, Z.; Liang, X.; Zhang, X.; Jia, Y.; Hu, M. A water-stable europium-MOF as a multifunctional luminescent sensor for some trivalent metal ions (Fe(3+), Cr(3+), Al(3+)), PO4(3-) ions, and nitroaromatic explosives. *Dalton Trans*. **2019**, *48*, 1786–1794.

[4] Chen, C.H.; Wang, X.S.; Li, L.; Huang, Y.B.; Cao, R. Highly selective sensing of Fe(3+) by an anionic metal-organic framework containing uncoordinated nitrogen and carboxylate oxygen sites. *Dalton Trans.* **2018**, *47*, 3452–3458.

[5] Hu, J.; Cheng, T.; Dong, S.; Zhou, C.; Huang, X.; Zhang, L. Multifunctional luminescent Cd (II)-based metal-organic framework material for highly selective and sensitive sensing 2,4,6-trinitrophenol (TNP) and Fe3+ cation. *Microporous Mesoporous Mater.* **2018**, *272*, 177–183.

[6] Ru, J.; Zhang, R.-F.; Li, X.-Y.; Wang, Y.-X.; Li, L.-L.; Ma, C.-L. Multi-responsive luminescent probes for Fe3+, Cr2O72- and acetylacetone with Cd-MOF based on tris(3 '-F-4 '-carboxybiphenyl)amine and trans-1,2-bis(4-pyridyl)ethene. *J. Solid State Chem.* **2022**, *307*, 122820.

[7] Chen, M.; Xu, W.-M.; Tian, J.-Y.; Cui, H.; Zhang, J.-X.; Liu, C.-S.; Du, M. A terbium(iii) lanthanide–organic framework as a platform for a recyclable multi-responsive luminescent sensor. *J. Mater. Chem. C* **2017**, *5*, 2015–2021.

[8] Li, F.-F.; Zhu, M.-L.; Lu, L.-P. A luminescent Cd(II)-based metal−organic framework for detection of Fe(III) ions in aqueous solution. *J. Solid State Chem.* **2018**, *261*, 31–36.

[9] Gai, Y.L.; Guo, Q.; Zhao, X.Y.; Chen, Y.; Liu, S.; Zhang, Y.; Zhuo, C.X.; Yao, C.; Xiong, K.C. Extremely stable europium-organic framework for luminescent sensing of Cr2O7(2-) and Fe(3+) in aqueous systems. *Dalton Trans.* **2018**, *47*, 12051–12055.

[10] Wang B.; Yang, Q.; Guo, C.; Sun, Y.; Xie, L.H. J.; Li, R. Stable Zr(IV)-Based Metal-Organic Frameworks with Predesigned Functionalized Ligands for Highly Selective Detection of Fe(III) Ions in Water. *ACS Appl. Mater. Interfaces* **2017**, *9*, 10286–10295.

[11] Guo, X.-Y.; Zhao, F.; Liu, J.-J.; Liu, Z.-L.; Wang, Y.-Q. An ultrastable zinc(ii)–organic framework as a recyclable multi-responsive luminescent sensor for Cr(iii), Cr(vi) and 4-nitrophenol in the aqueous phase with high selectivity and sensitivity. *J. Mater. Chem. A* **2017**, *5*, 20035–20043.

[12] Jin, H.; Xu, J.; Zhang, L.; Ma, B.; Shi, X.; Fan, Y.; Wang, L. Multi-responsive luminescent sensor based on Zn (II) metal-organic framework for selective sensing of Cr(III), Cr(VI) ions and p-nitrotolune. *J. Solid State Chem.* **2018**, *268*, 168–174.

[13] Lv, R.; Wang, J.; Zhang, Y.; Li, H.; Yang, L.; Liao, S.; Gu, W.; Liu, X. An amino-decorated dual-functional metal–organic framework for highly selective sensing of Cr(iii) and Cr(vi) ions and detection of nitroaromatic explosives. *J. Mater. Chem. A* **2016**, *4*, 15494–15500.

[14] Liu, J.; Ji, G.; Xiao, J.; Liu, Z. Ultrastable 1D Europium Complex for Simultaneous and Quantitative Sensing of Cr(III) and Cr(VI) Ions in Aqueous Solution with High Selectivity and Sensitivity. *Inorg. Chem.* **2017**, *56*, 4197–4205.

[15] Meng, X.; Wei, M.J.; Wang, H.N.; Zang, H.Y.; Zhou, Z.Y. Multifunctional luminescent Zn(ii)-based metal-organic framework for high proton-conductivity and detection of Cr(3+) ions in the presence of mixed metal ions. *Dalton Trans.* **2018**, *47*, 1383–1387.

[16] Liang, X.; Jia, Y.; Zhan, Z.; Hu, M. A highly selective multifunctional Zn-coordination polymer sensor for detection of Cr (III), Cr (VI) ion, and TNP molecule. *Appl. Organomet. Chem.* **2019**, *33*, e4988.

[17] Li, L.; Chen, F.-F.; Pan, J.; Zhong, S.; Li, L.; Yu, Y. Amino-functionalized YF3:Eu3+ nanoparticles: A selective two-in-one fluorescent probe for Cr(III) and Cr(VI) detection. *J. Lumin.* 2020, *226*, 117440.

[18] Vashistha, N.; Chandra, A.; Singh, M. HSA functionalized Gd2O3:Eu3+ nanoparticles as an MRI contrast agent and a potential luminescent probe for Fe3+, Cr3+, and Cu2+ detection in water. *New J. Chem.* **2020**, *44*, 14211–14227.

[19] Wang, C.; Xu, J.; Li, H.; Zhao, W. Tunable multicolour S/N co-doped carbon quantum dots synthesized from waste foam and application to detection of Cr(3+) ions. *Luminescence* **2020**, *35*, 1373–1383.

[20] Dutta, B.; Jana, R.; Bhanja, A.K.; Ray, P.P.; Sinha, C.; Mir, M.H. Supramolecular Aggregate of Cadmium(II)-Based One-Dimensional Coordination Polymer for Device Fabrication and Sensor Application. *Inorg. Chem.* **2019**, *58*, 2686–2694.

[21] Liu, W.; Huang, X.; Xu, C.; Chen, C.; Yang, L.; Dou, W.; Chen, W.; Yang, H.; Liu, W. A Multi-responsive Regenerable Europium-Organic Framework Luminescent Sensor for Fe(3+) , Cr(VI) Anions, and Picric Acid. *Chemistry* **2016**, 22, 18769–18776.

[22] Li, G.P.; Liu, G.; Li, Y.Z.; Hou, L.; Wang, Y.Y.; Zhu, Z. Uncommon Pyrazoyl-Carboxyl Bifunctional Ligand-Based Microporous Lanthanide Systems: Sorption and Luminescent Sensing Properties. *Inorg. Chem.* **2016**, *55* , 3952–3959.

[23] Wen, G.X.; Han, M.L.; Wu, X.Q.; Wu, Y.P.; Dong, W.W.; Zhao, J.; Li, D.S.; Ma, L.F. A multi-responsive luminescent sensor based on a super-stable sandwich-type terbium(iii)-organic framework. *Dalton Trans.* **2016**, *45*, 15492–15499.

[24] Xu, T.-Y.; Li, J.-M.; Han, Y.-H.; Wang, A.-R.; He, K.-H.; Shi, Z.-F. A new 3D four-fold interpenetrated dia-like luminescent Zn(ii)-based metal–organic framework: the sensitive detection of Fe3+, Cr2O72−, and CrO42− in water, and nitrobenzene in ethanol. *New J. Chem.* **2020**, *44*, 4011–4022.

[25] Parmar, B.; Rachuri, Y.; Bisht, K.K.; Laiya, R.; Suresh, E. Mechanochemical and Conventional Synthesis of Zn(II)/Cd(II) Luminescent Coordination Polymers: Dual Sensing Probe for Selective Detection of Chromate Anions and TNP in Aqueous Phase. *Inorg. Chem*. **2017**, *56*, 2627–2638.
